# Healthy clocks, healthy body, healthy mind

**DOI:** 10.1016/j.tcb.2009.10.005

**Published:** 2010-01

**Authors:** Akhilesh B. Reddy, John S. O’Neill

**Affiliations:** Department of Clinical Neurosciences, University of Cambridge Metabolic Research Laboratories, Institute of Metabolic Science, Cambridge CB2 OQQ, UK

## Abstract

Circadian rhythms permeate mammalian biology. They are manifested in the temporal organisation of behavioural, physiological, cellular and neuronal processes. Whereas it has been shown recently that these ∼24-hour cycles are intrinsic to the cell and persist *in vitro*, internal synchrony in mammals is largely governed by the hypothalamic suprachiasmatic nuclei that facilitate anticipation of, and adaptation to, the solar cycle. Our timekeeping mechanism is deeply embedded in cell function and is modelled as a network of transcriptional and/or post-translational feedback loops. Concurrent with this, we are beginning to understand how this ancient timekeeper interacts with myriad cell systems, including signal transduction cascades and the cell cycle, and thus impacts on disease. An exemplary area where this knowledge is rapidly expanding and contributing to novel therapies is cancer, where the *Period* genes have been identified as tumour suppressors. In more complex disorders, where aetiology remains controversial, interactions with the clockwork are only now starting to be appreciated.

## Introduction

Circadian (*circa*-, ‘approximately’; -*diem,* ‘day’) rhythms are a fundamental property of living cells. When held in temporal isolation, organisms from unicells to humans exhibit behavioural and physiological rhythms that persist with a period of approximately 24 h [1]. These rhythms are driven by biological clocks that have two essential features. First, their free-running period of ∼24 h is temperature-compensated: clocks do not run slower at lower temperatures or speed up when it is hot – a remarkable and necessary feat of biochemical engineering. Second, they can synchronise to temporally relevant stimuli such as light, temperature or feeding schedules, and thus their definition of internal time becomes predictive of external (solar) time [2]. Entrained in this way, clocks confer selective advantages to organisms by facilitating anticipation of, and thereby adaptation to, the alternating day–night cycle as well as temporally segregating mutually antagonistic processes that might otherwise result in a futile cycle – for example, glycolysis (day) and gluconeogenesis (night) in hepatocytes [3]. The competitive value of circadian clocks has been demonstrated in prokaryotes and higher plants [4,5], and disturbance of circadian timing in humans, as seen in rotational shift workers for example, carries significant long-term health costs [6].

Rhythmic regulation of behaviour and physiology results from the circadian modulation of diverse processes and pathways, and therefore interactions between the clock and health are necessarily pleiotropic in nature. Two clear trends can be identified however. Namely, that organisms whose internal clocks are synchronised with the external environment are healthier (more adept at dealing with environmental challenge) [7], and that genetic or acute lesions affecting timekeeping reduce temporal homeostasis with concomitant health consequences, albeit often indirectly [8]. For example, in the context of cancer, it has been shown that the circadian cycle gates cell division [9], and thus loss of cellular rhythmicity might be expected to correlate with increased cellular transformation. Indeed, a number of canonical clock genes have been identified independently as tumour suppressors; for example, *Bmal1* [10]. When otherwise healthy humans or rodents are repeatedly desynchronised from the external environment, however, an increased cancer risk and reduced longevity is also observed [11,12].

## Healthy clockwork = healthy body and mind

The human body is a cyclical machine, and circadian variation in physical and cognitive performance is readily observable at both the individual and population levels [13,14] (Box 1, panels A,B). These behavioural outputs stem from circadian regulation of neuronal, physiological and endocrine function; examples include rhythms in core body temperature, heart rate, and in cortisol and melatonin secretion [15,16]. Indeed, the majority of body and cell functions, where studied, appear to have some circadian component. For example, elements of both the adaptive and innate immune system are subject to circadian regulation [17], as is the severity of many disease states including myocardial infarction [18] and depression [19]. Indeed, more than 20% of gene expression in a given tissue has been estimated to be under circadian regulation at either the transcriptional or protein level, with further circadian regulation being evident through post-translational protein modification [20]. There is no doubt, therefore, that our bodies are temporally orchestrated by the clockwork, but what are the consequences when our internal clocks are disrupted, or become misaligned with the external environment, as occurs in jet-lag and in shift work?

There is mounting evidence to suggest that long-term disruption of rhythmic behaviour correlates with disease states, leading to profound implications for healthcare in the future [21]. Interestingly, diseases such as ischaemic stroke, that share risk factors with cardiovascular disease, have similarly been found to occur more frequently in female long-term shift workers [22]. Moreover, in this cohort there appears to be a clear link between breast cancer risk and long-term shift working [23,24], and this is being taken seriously by a number of governments in view of increasing litigation in this area [25]. A number of additional studies have found that chronic shift work is significantly associated with an increased risk of colorectal, endometrial and prostate cancers [26]. Similarly, recent work highlights that cardiovascular and metabolic dysfunction (glucose intolerance) occur in situations analogous to rotational shift work [27], meaning that how we work could have consequences for the development of such conditions. Obesity, diabetes and related metabolic syndromes are on the increase globally, and novel ways to combat them are needed. In this context, the observation that ‘statins’ are most efficacious when administered during subjective night has been known anecdotally for years [28], but when coupled with a recent report that mice fed at the ‘wrong time’ of day (i.e. when they are supposed to be sleeping) gain weight more rapidly than littermates fed at the ‘right time’ [29], one may infer that attempts to cure or prevent diseased states in humans will be hindered unless the circadian context of treatment is considered. There thus exists a clear need to understand the molecular mechanisms that sustain our clockwork and to elucidate its interactions with other biological systems.

## From rhythmic behaviour to molecular events

Our view of circadian rhythms has changed immensely over the last decade or so. For many years the consensus view was that mammalian timekeeping function was highly centralised within a so-called master clock – the suprachiasmatic nuclei (SCN) that integrate relevant environmental cues (photic and non-photic) and signal timing information to peripheral tissues through via neuronal efferents and diffusible factors [30–32] (Box 1, panel C). This bilateral structure comprises approximately 10,000 neurons and resides in the basal hypothalamus, above the optic nerve crossing (chiasm), and is ideally situated to be entrained by ambient lighting cues relayed from a sub-population of intrinsically photosensitive retinal ganglion cells [33]. The SCN was shown to be indispensable for coherent behavioural rhythmicity in mammals in the 1970s. When it is ablated in mice, or damaged in humans, behavioural cycles including sleep and wakefulness become arrhythmic or disorganised [31,34]. Indeed, intrinsic rhythmic activity in the SCN is so robust that rhythms have been shown to persist for months in organotypic slices *in vitro* [35]. Moreover, mutations affecting the ability of the SCN to function as an assemblage (e.g. in VPAC2 receptor knockout mice), and mutations that either ‘accelerate’ the clockwork (e.g. homozygous *Tau* mutant mice or hamsters exhibit a significantly shorter free-running periodicity of ∼21 hours) or slow it down (e.g. *Fbxl3* mutant mice, with an endogenous periodicity of ∼28 hours), all directly impact on rhythmic behaviour, further illustrating the SCN's pivotal orchestrating role *in vivo* [36–41].

Significantly, however, in the last 5 years it has been shown beyond doubt that whereas the SCN clearly plays a key role in synchronising rhythms across the body's various tissues, cells within those tissues themselves exhibit self-sustained rhythms in gene expression that also persist in culture [35,42] (Box 1, panel D). Indeed, in mice with a conditionally inactive clock in the liver, the number of transcripts that continued to oscillate and were therefore driven directly by humoral factors was only ∼10% compared to wild-type controls [43,44], implying that the majority of oscillating transcripts are reliant upon cell-intrinsic mechanisms. Moreover, in addition to serum factors and pharmacological cues, very mild 24-hour temperature cycles (that mimic circadian variation in body temperature) are sufficient to entrain the phase of fibroblasts *in vitro*, implying that peripheral tissues are also competent to be stably entrained to timing cues in the absence of SCN signalling [9,45–49]. Finally, when entrained via feeding or pharmacological cues, it has been shown that the SCN, and even several identified ‘clock genes’, are dispensable for rhythmic behaviour [50,51]. Thus, the generic mechanism that sustains intracellular rhythms in all tissues has become a major focus for circadian research (Box 1 overviews the current molecular model and its relation to higher-order biological structures and behaviour).

The early successes of molecular approaches to identifying clock components revealed a number of transcription factors (Per1–3, Cry1,2, Bmal1, Clock etc.) that appeared to act in a day-long auto-regulatory negative feedback loop, regulating the rhythmic expression of many clock-controlled genes in a tissue-specific manner [42,52]. More recently, however, several rhythmic outputs from this core loop have been shown, in turn, to also feedback into it – rendering the principle of a core mechanism increasingly semantic. Strikingly, these additional loops include not only additional transcription factors (e.g. Dec1,2, Rev-erbα) [53] but also several ubiquitous pathways that are heavily implicated in other cellular processes. For example, AMPK is involved in cellular energy homeostasis but was recently shown to also display rhythmic activity and localisation, and regulate the stability of Cry1 [54]. Similarly, cAMP signalling is an essential signal transduction pathway, but is also described as a core clock component, governing the period, amplitude and phase of rhythms in gene expression [55]. In addition, the NAD/NADH redox balance, so crucial to cellular metabolism, has been shown to have reciprocal regulation with the core clock mechanism (see below) [56]. Whether the participation of these essential cellular systems reflects an inbuilt distributed functional redundancy or a deeper biological truth remains to be seen. Certainly, our picture of what constitutes the minimal cellular timing architecture has become somewhat cluttered of late, and our drive to separate cause from effect dictates that an quantitative assessment of the relevant functional contribution of each putative component to timekeeping is overdue, especially because transfer of this understanding to clinical relevance is the foremost objective.

## The clock and cancer: a tale of two cycles

Possible interactions between the cell cycle and the clockwork have been known for some time [57–61], in the sense that the clock gates cell division to specific circadian phases. From an evolutionary perspective this is intuitive because DNA synthesis and replication performed at night are not exposed to harmful UV radiation that might otherwise have deleterious effects on replicative fidelity. In mammals, there is a vast body of evidence, spanning more than two decades, that has defined circadian variation in mitotic indices in a multitude of tissues including oral mucosa, skin, intestinal epithelium, and bone marrow [62]. Only recently, however, have the mechanisms underlying this link been revealed and, lately, translated to the clinical oncology arena [62–65].

**Figure 1:**
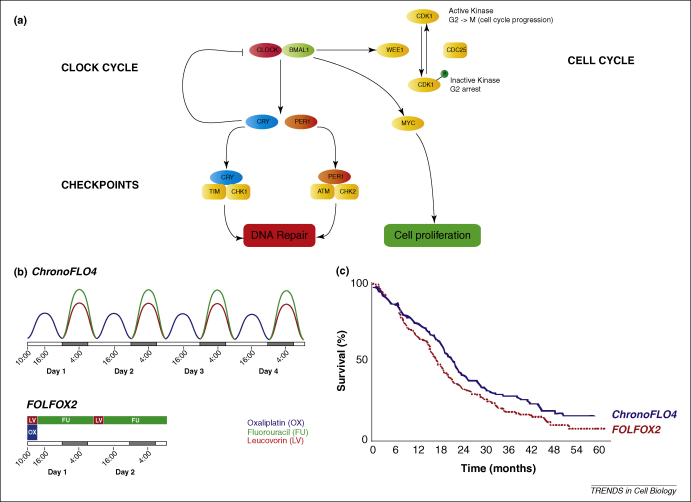
Clocks, cancer and the cell cycle. **(A)** The circadian system is linked to the cell-division cycle through circadian control of gene expression and post-translational mechanisms. Transcription of the myelocytomatosis (*Myc*) oncogene and of *Wee1* is circadian and this appears to be a direct target of the CLOCK:BMAL1 complex. The expression of *Wee1* is coregulated with that of period homologue genes (*Per*) and the entry of the cell cycle into M phase is suppressed during the daytime when the transcription of *Per* (and *Wee1*) is high. In addition, the PER1 protein interacts with the checkpoint proteins ataxia telangiectasia mutated (ATM) and checkpoint kinase 2 (CHK2), whereas related work has linked the timeless (TIM) and cryptochrome (CRY) proteins with CHK1. Activation of the DNA-damage pathway can also reset the phase of the circadian clock. CDC25, cell division cycle 25; CDK1, cyclin-dependant kinase 1 (adapted from Ref. [2]). **(B)** Treatment schedules combining oxaliplatin (Oxal), fluorouracil (FU), and leucovorin (LV) administered as a chronomodulated infusion over 4 days (chronoFLO4) or as a conventional infusion over 2 days (FOLFOX2). The abscissa represents alternating spans of 8 hours of darkness, corresponding to the average rest span at night, and 16 hours of light, corresponding to the average duration of daytime wakefulness, over the course of chemotherapy delivery. **(C)** Overall survival curve for men, indicating a superior survival at 5 years in the chronomodulated (chronoFLO4) chemotherapy group (adapted from Ref. [91]).

Beyond the observation that circadian disruption shortens survival and accelerates malignant growth, insights broadly split into two categories: clock genes as tumour supressors or oncogenes and therefore putative prognostic indicators, and chronotherapeutic applications resulting from circadian regulation of cell proliferation and detoxification pathways. In the case of the former, defects in core clock components (such as *Period 1* and *2*; putative tumour suppressors) have been shown to be involved in the response to radiation-induced DNA damage, and hence the propensity for tumour formation *in vivo* [66,67]. In addition, the core clock components Clock:Bmal1, and the close homologue Npas2, have been shown to protect against chemical and radiation-induced damage [68–70], Indeed, Npas2 is being investigated as a prognostic biomarker for breast cancer [71]. By contrast, loss of the putative oncogene *Cryptochrome* significantly reduces cancer risk in *p53* mutant mice [72] and *Cryptochrome2* has further been implicated in the development of non-Hodgkin's Lymphoma [73]. Moreover, a variety of classical cell cycle and/or proliferation genes (such as *c-Myc* and *Wee1*) have been shown to be under direct clock control, and their expression (driven by Clock:Bmal1 complexes via E-box elements in their regulatory regions) effectively gates the division of non-transformed cells to specific circadian phases [62,64,66]. Other well-recognised cell-cycle regulators (e.g. *Cyclin D1* and *Mdm-2* and *Gadd45α*) are likely to be controlled indirectly [66] but, strikingly, the interaction is bidirectional in that DNA damage can reset the phase of the clockwork (Figure 1A) [74,75], presumably because repair to genetic material has to take priority over a system that contributes to cellular homeostasis in the longer term.

In tumours themselves, consistent changes in the expression of clock genes (e.g. *Per1–3*) have been demonstrated, as well as changes in the methylation state of their regulatory regions in various tissues including breast, liver and endometrial cancers [76–78]. The extent to which cancer cells are able to dispense with normal circadian gating of cell division is however unclear [79], and it could well be that tumours instead become insensitive to peripheral cues and, in effect, free run [80,81]. Demonstrably though, when the daily timing of animals is upset using conditions mimicking jet-lag or shift work, implanted malignant tumours grow more rapidly than in unperturbed controls [81], correlating well with reports of shortened survival in cancer patients with abnormal rhythms [82–84] and supported by epidemiological meta-analyses of tumour induction [85]. Indeed, several clock gene polymorphisms are being actively investigated as cancer risk factors [73,86]. Thus there is compelling evidence that ‘clock mechanisms’ are also inexorably tied up in cell proliferation and its control at the DNA (epigenetic), RNA and protein levels.

How is this new knowledge being applied clinically? Changes in clock gene expression might find use as biomarkers for cancers [73], but the most exciting avenue has been the development of ‘chronotherapy’ for cancer – using medications at times when they will be most effective on cancer cells, while simultaneously minimising side effects [65,87]. Circadian dosing time influences the extent of toxicity of more than thirty anticancer drugs, and it has been shown in animal models that survival rate varies by at least 50% depending on when a ‘lethal dose’ of drug is given [15]. This might well be because cellular repair mechanisms, such as nucleotide excision, are subject to circadian regulation [88], as are many key genes associated with xenobiotic metabolism and transport [89]. Even more strikingly though, the administration of a drug at a circadian time when it is best tolerated usually achieves the best anti-tumour activity [90]. This knowledge is being applied increasingly in clinical trials. For example, Giacchetti and colleagues recently conducted a Phase III trial comparing ‘chronomodulated’ administration of fluorouracil (5-FU), leucovorin, and oxaliplatin against the standard regime in patients with newly diagnosed metastatic colorectal cancer (Figure 1B,C) [91]. There was a significant survival advantage of the chronomodulated regime, but this was interestingly only confined to men, whereas women fared better on conventional delivery, highlighting the need for further such studies. Moreover, rodent studies using the drug seliciclib, a cyclin-dependent kinase inhibitor, showed that drug treatment during subjective day reduced tumour growth by more than 50% compared with subjective night [92], and that this increased reduction apparently resulted from restoration of normally phased clock gene expression patterns in the tumours [93].

## The ageing clock: neurodegeneration and rhythmic behaviour

At the behavioural level, it is well established that aged organisms behave less rhythmically [12]. Indeed, loss of regular sleeping patterns in humans is the one of the prime motivations for institutionalisation of the elderly [94]. Furthermore, this loss of daily rhythms in sleep–wake activity has been speculated to contribute to the onset of neurodegenerative disorders and could be considered a pre-symptomatic correlate [94–96]. Conversely, behaviourally arrhythmic animals exhibit accelerated ageing – for example the *Bmal1*^−/−^ mouse exhibits loss of behavioural and molecular circadian rhythms because it lacks Bmal1-driven rhythmic transcription of downstream clock genes from their cognate E-box promoter sequences [97]. These mice also exhibit reduced body weight and show mortality at around six months in the absence of major pathological changes in their major organ systems [98]. Thus, whereas a bidirectional interaction between the circadian clock and the ageing process has long been suspected, until recently there was little molecular evidence to substantiate it. Many theories of cellular senescence, however, posit impaired redox regulation at their core [99], and so the observation that Npas2, a functional homologue of Clock, could also be affected by the energy/redox status of the cell, and thus potentially influence circadian transcription, was a surprise to the circadian field [100,101]. More specifically, it is the redox balance of nicotinamide adenine dinucleotide (NAD) cofactors that influence this: the reduced forms NAD(H) and NADP(H) strongly enhance DNA binding of the Clock:Bmal1 and Npas2:Bmal1 heterodimers, whereas the oxidized forms inhibit binding [101]. Additional redox-sensing ligands (e.g. heme) have subsequently been identified in the regulation of *Per2* and *Rev-Erbα* activity [102,103]; other recent work in this area has produced further insights into this core biochemical machinery and has highlighted a role for the deacetylase SIRT1 (homologous to Sir2 in yeast) in this process [104–107]. SIRT1 has broad biological functions in growth regulation, stress response, tumourigenesis, endocrine signalling, and in extending lifespan [108]. In the present context SIRT1 appears to counteract the transcription-activating function of Clock protein that was recently shown to exhibit histone acetyl-transferase activity, and thus controls chromatin remodelling around target genes. SIRT1 therefore facilitates repressive chromatin structures in anti-phase to Clock:Bmal1 because, crucially, SIRT1 activity is NAD-dependent and therefore sensitive to the redox balance of the cell, that is itself subject to circadian regulation through modulation of NAD synthesis pathways [104]. It remains to be seen how the clockwork and cell ageing interact at the molecular level to produce the whole-animal effects observed during senescence.

**Figure 2:**
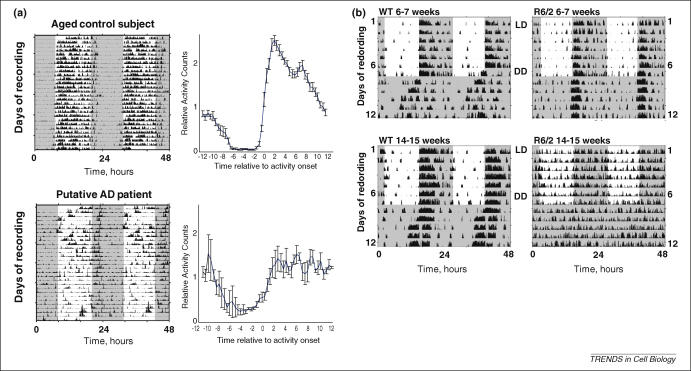
The clockwork and neurodegenerative disorders. **(A)** Representative actograms from healthy control (top) and moderately demented (bottom) patients. Data from 28 consecutive days are double-plotted on a 48-hour time base for clarity. Group daily activity profiles (plotted as means ± SEM) and moderately demented subjects are shown to the right; adapted from Ref. [94]. **(B)** Progressive changes in the activity–rest cycles of control mice (left) and R6/2 mice (right) before they develop motor and/or cognitive symptoms (6–7 weeks) and after they exhibit overt signs of disease (14–15 weeks). LD, light–dark cycle; DD, constant dim red light.

An additional correlate of ageing in humans is the increased incidence of neurodegenerative disorders. Whereas it has been known for many years that poor sleep health is associated with a range of neuronal diseases, the links between circadian dysfunction and Alzheimer's or Huntington's disease, for example, has only recently begun to be studied in detail. Disturbed sleep cycles are the principal cause of institutionalisation in dementia, and therefore represent a major clinical problem [94]. Certainly, disturbances in the activity–rest cycles of patients with certain dementias (most notably Alzheimer-type and fronto-temporal dementia) have suggested that circadian rhythm disturbance might contribute to the disease process, or be a reflection of it [94,95,109] (Figure 2A). Recently, there has been much interest in the disturbance of circadian rhythms in Huntington's disease. Disintegration of sleep–wake cycles and circadian gene expression across the brain occurs in the R6/2 mouse model of Huntington's disease, and this disturbance becomes more profound as the animal's brain degenerates (Figure 2B) (Ref. [110]). More impressively, cognitive decline and dysfunctional circadian gene expression can be reversed by imposing a daily cycle of sleep in R6/2 mice with the benzodiazepine alprazolam, and this leads to a significant improvement in survival of the diseased mice [111,112]. This latest evidence suggests therefore that circadian and sleep disruption contribute to the neuronal damage that occurs in Huntington's disease, and that targeting the clockwork could be a novel way to combat this archetypal genetic disorder, with obvious implications for other related neurodegenerative conditions.

## Concluding Remarks

Our emerging view of circadian clocks at the cellular and molecular levels revolutionises the way we view the physiological processes that occur in our bodies. Whether it is the regulation of daily cell metabolism, the cell division cycle or the modulation of mood and neurological function, the circadian clockwork is hard-wired into these processes. A key example of this is provided by the Period proteins that are involved in tumour suppression. Understanding the programmes that mould tissue-specific gene and protein expression is beginning to lead to insights into how we can best use this knowledge to direct existing therapies and interventions. This is being applied currently in oncology, but endocrinologists in the future are likely to use drugs modulating clock outputs to treat obesity and its sequelae, including diabetes and metabolic syndrome. Nuclear hormone receptors are emerging targets for drug therapy and could link disease states in multiple tissues to tractable therapeutics in the future. Current barriers to translating basic findings to the clinic can only be addressed with further research into the complex interactions between the distributed network of clocks around the body and how they are synchronised (Box 2). Moreover, new discoveries will propel the development of completely novel therapeutic regimens.

## Figures and Tables

**Figure I:**
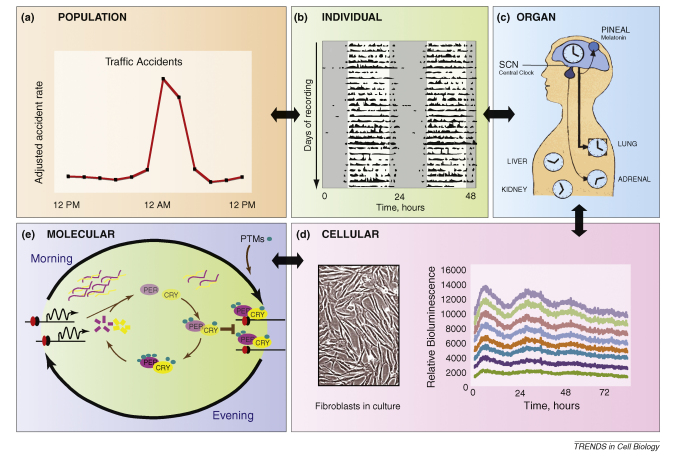
Montage showing the hierarchical nature of circadian rhythms.

## Box 1 The molecular clockwork

The montage in Figure I shows the hierarchical nature of circadian rhythms from **(A)** Population (the incidence of road traffic accidents varies across the day, adapted from Ref. [113]); to **(B)** Individual behavioural activity shown on a double-plotted actigram; to **(C)** Physiology and/or Organ systems that, although at different phases of gene expression, are maintained in a stable phase relationship with one another through signalling from the SCN; to **(D)** Individual cells, for example fibroblast rhythms that can be observed in real-time through bioluminescent promoter-fusion reporters; to **(E)** Molecular oscillations where current models of timekeeping revolve around autoregulatory, inter-linked transcriptional and post-translational feedback loops in which, at the start of circadian daytime, the positive factors Clock (red) and Bmal1 (black) activate the expression of the negative regulators Period (Per1,2,3, purple) and Cryptochrome (Cry1,2; yellow) via E-box regulatory sequences. With the progression of circadian daytime the abundance of Per/Cry complexes in the nucleus increases. On progressing into circadian night-time these complexes start to suppress E-box activation, closing the negative feedback loop. Thus the dynamic balance changes and, because the rate of protein degradation exceeds *de novo* synthesis, Per/Cry complexes disappear from the nucleus. By the end of the circadian night the negative regulation is lifted and a new circadian day is initiated [114]. Thus the dynamics of translation, intracellular trafficking, complex formation and covalent post-translational modifications (PTMs, green ovals), such as phosphorylation and acetylation, and ultimately proteasomal degradation, will contribute to the pace and stability of timekeeping. In particular the activity of ubiquitous cellular kinases such as casein kinase 1/2, glycogen synthase kinase 3 and AMP kinase have been shown to be intimately connected with clock protein progression through the cycle [115], In addition, promoter chromatin structure cycles through open and repressive states in response to the NAD+/NADH redox balance of the cell through the action of histone acetyl-transferases (e.g. Clock, NADH-dependent) and histone deacetylases (e.g. SIRT1, NAD+-dependent; not shown). Stability and contrast enhancement are also conferred by auxiliary feedback loops, such as those involving the orphan nuclear receptors RORA and Rev-Erbα (not shown) that are activated by Clock:Bmal1 and have, respectively, positive and negative actions on Bmal1 via RORE sequences [116]. By regulating the expression of ‘clock-controlled genes’ that carry E-box and RORE sequences, but are not involved in these feedback loops, the daily waxing and waning of Per, Cry, Bmal1, RORA and Rev-Erbα clock proteins is able to impose a daily order on cell activity, ultimately generating the behaviour and physiology of the organism [20,34,117,118]. Complicating this picture, however, are recent reports that many clock genes are dispensable for cellular rhythmicity, whereas diverse cellular mechanisms, such as microRNA-mediated repression, cAMP signalling and redox metabolism that are under clock control also feedback into the so-called ‘central’ clock mechanism [55,119,120]. Given that the cellular oscillator has also recently been shown to be remarkably robust to gross inhibition of global transcriptional activity [121], it is presently unclear to what extent it is helpful to consider the transcriptional feedback circuitry in isolation from its wider cellular context [32,122].

## Box 2 Future questions

1. Is the molecular clock inexorably linked to the rest of the cell's machinery?
2. How is daily timing (and its dysfunction) linked to ageing?
3. Are changes seen in neurodegeration a cause or consequence of clock dysfunction?
4. Can resynchronisation of clocks correct metabolic derangements?
5. Will drugs in the future be delivered according to the time of day, and be individually tailored for a person's ‘chronotype’?
